# Increased Hemichannel Activity Displayed by a Connexin43 Mutation Causing a Familial Connexinopathy Exhibiting Hypotrichosis with Follicular Keratosis and Hyperostosis

**DOI:** 10.3390/ijms24032222

**Published:** 2023-01-22

**Authors:** Olivia E. Crouthamel, Leping Li, Michael T. Dilluvio, Thomas W. White

**Affiliations:** 1 MS Program in Biomedical Sciences (Physiology and Biophysics Track), Stony Brook University, Stony Brook, NY 11794, USA; 2 Department of Physiology and Biophysics, Stony Brook University School of Medicine, Stony Brook, NY 11794, USA

**Keywords:** *GJA1*, connexin, hemichannel, gap junction, genetic disease

## Abstract

Mutations in the *GJA1* gene that encodes connexin43 (Cx43) cause several rare genetic disorders, including diseases affecting the epidermis. Here, we examined the in vitro functional consequences of a Cx43 mutation, Cx43-G38E, linked to a novel human phenotype of hypotrichosis, follicular keratosis and hyperostosis. We found that Cx43-G38E was efficiently translated in *Xenopus* oocytes and localized to gap junction plaques in transfected HeLa cells. Cx43-G38E formed functional gap junction channels with the same efficiency as wild-type Cx43 in *Xenopus* oocytes, although voltage gating of the gap junction channels was altered. Notably, Cx43-G38E significantly increased membrane current flow through the formation of active hemichannels when compared to wild-type Cx43. These data demonstrate the association of increased hemichannel activity to a connexin mutation linked to a skeletal-cutaneous phenotype, suggesting that augmented hemichannel activity could play a role in skin and skeletal disorders caused by human Cx43 mutations.

## 1. Introduction

Connexins were first identified as the subunit proteins of the intercellular channels present in vertebrate gap junctions [1,2]. In addition to forming gap junction channels, some connexins were later shown to be able to also function as membrane channels, called hemichannels, directly linking the cytoplasm to the extracellular space [3,4,5]. Since this discovery, determining roles for connexin hemichannels in normal physiology has remained an active area of research, as has the linkage of aberrant hemichannel activity to specific pathophysiology in a variety of tissues [6,7,8]. Mutations in numerous connexin genes have been linked to many genetic diseases [9,10,11]. In several of these conditions, alteration of connexin hemichannel activity has been implicated as a key part of the pathological mechanism [8,12,13,14,15,16,17].

For example, mutations in the human *GJA1* gene encoding Cx43 can cause a number of distinct rare genetic disorders. The most frequently resulting disease is oculodentodigital dysplasia (ODDD), which is usually a dominant disorder, but can also be inherited as a recessive disease [9,18,19,20]. ODDD patients have syndactaly, microphthalmia and craniofacial and dental abnormalities. Some patients can also present with microcephaly, hypotrichosis and brittle nails [18,21]. Occasionally, patients can also develop skin abnormalities, including palmoplantar keratoderma [22]. Mutations in Cx43 can also cause recessive cranio-metaphyseal dysplasia (CMDR) [23], a disease characterized by thickening of the skull bones and abnormalities at the ends of long bones of the limbs. The ocular, digital and dental phenotypes of ODDD are not present in individuals with CMDR [24,25]. Finally, Cx43 mutations underlie dominant skin-limited epidermal disorders such as erythro-keratodermia variabilis et progressive (EKVP) [26], or palmoplantar keratoderma and congenital alopecia-1 (PPKCA1) [27]. Hyperkeratosis, erythematous patches, prominent white lunulae and periorificial darkening characterize EKVP caused by Cx43 mutations. In about half of the patients, palmoplantar keratoderma is also present [26,28]. PPKCA1 patients display severe hyperkeratosis, congenital alopecia and leukonychia [29]. Thus, human genetic diseases caused by mutations in Cx43 often result in abnormalities of bone and skin.

Increased activity of Cx43 hemichannels has been identified as a common functional consequence of the Cx43 mutations that cause skin limited disorders such as EKVP and PPKCA1 [27,30,31]. This is consistent with aberrant hemichannel activity associated with a majority of the epidermal disorders caused by mutations in other connexin genes [12,32,33,34,35]. In the case of ODDD, Cx43 mutations are thought to contribute to the complex pathology through at least ten different mechanisms [19,36], including increased hemichannel activity in some cases [37,38]. To our knowledge, the functional consequences of Cx43 mutations linked to CMDR have not been determined. Therefore, altered hemichannel activity is thought to play a pathological role in some of the human disorders linked to Cx43 mutations, particularly those with skin involvement.

Here, we have examined the functional consequences of a Cx43 mutation, Cx43-G38E, linked to a novel human phenotype of hypotrichosis, follicular keratosis and hyperostosis [39]. We found that Cx43-G38E was efficiently translated in *Xenopus* oocytes and localized to gap junction plaques in transfected HeLa cells. Cx43-G38E showed significantly increased hemichannel activity compared to wild-type Cx43, and also formed functional gap junction channels with altered voltage gating. Together, these data demonstrate an additional finding of increased hemichannel activity in a human Cx43 mutation linked to a skeletal-cutaneous phenotype.

## 2. Results

### 2.1. Cx43-G38E Is Translated in Xenopus Oocytes at Higher Levels Than Wild-Type Cx43

**Figure 1:**
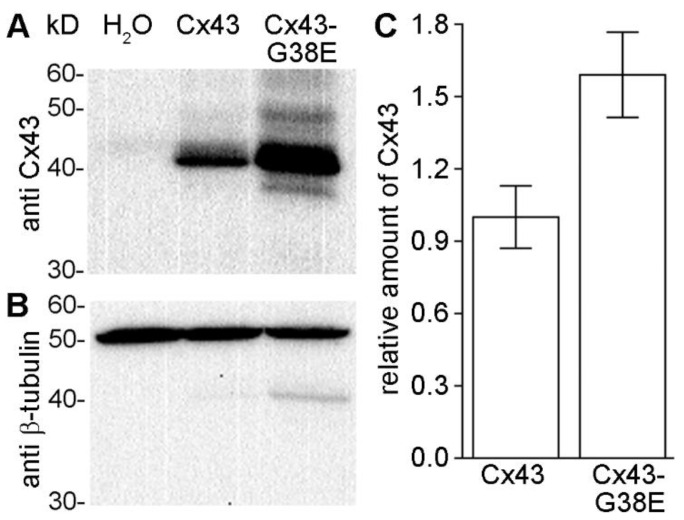
Expression of wild-type and mutant connexins in *Xenopus* oocytes. (**A**) Western blots probed with an anti-Cx43 antibody showed a prominent 43 kD band in protein samples derived from oocytes injected with wild-type Cx43, or mutant Cx43-G38E RNAs. Oocytes injected with water as a negative control showed no Cx43 signal. (**B**) Re-probing of the blots with an antibody against β-tubulin showed confirmed equivalent loading of total protein in all three samples. (**C**) Analysis of band density revealed that mutant Cx43-G38E was translated at levels ~1.6-fold higher than wild-type Cx43 (*p* < 0.05, *t*-test) Data are plotted as the mean ± SE and are derived from five independent experiments.

The levels of protein expression of wild-type Cx43 and mutant Cx43-G38E were assessed by western blotting of membrane preparations from *Xenopus* oocytes that had been injected with equal amounts of wild-type or mutant RNA. Immunoblotting with anti-Cx43 antibodies detected major 43 kDa bands in the lanes loaded from oocytes injected with either wild-type Cx43, or Cx43-G38E RNAs. In water injected control cells, the antibody detected no Cx43 band (Figure 1A). Stripping and re-probing of the blots with anti-β-tubulin antibodies showed that tubulin protein was present at equivalent levels in all three samples, confirming equal protein loading (Figure 1B). Densitometry revealed that mutant Cx43-G38E proteins were expressed at levels ~1.6-fold higher than wild-type Cx43 on average (*p* < 0.05, n = 5). These data suggest that both wild-type Cx43 and Cx43-G38E were efficiently expressed in RNA injected *Xenopus* oocytes. However, they also showed that injection of identical amounts of wild-type Cx43 and mutant Cx43-G38E RNAs resulted in higher levels of mutant protein synthesis when compared to wild-type.

### 2.2. The Cx43-G38E Mutation Displays Increased Hemichannel Activity

**Figure 2:**
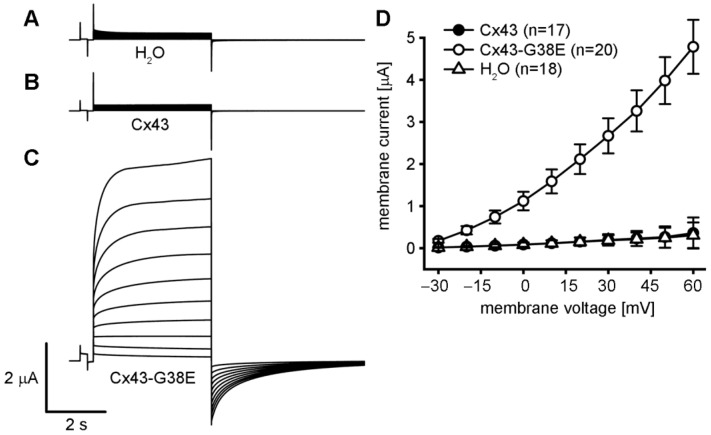
The Cx43-G38E mutation forms active connexin hemichannels. Single oocytes were clamped at –40 mV and subjected to voltage pulses ranging from –30 to +60 mV in 10 mV steps. (**A**) H_2_O and (**B**) wild-type Cx43 injected oocytes displayed negligible membrane currents. (**C**) Cx43-G38E expressing oocytes had much larger hemichannel currents than wild-type Cx43. (**D**) Peak currents from each pulse were plotted as a function of membrane voltage. Peak currents in wild-type Cx43 (filled circles), or H_2_O injected (open triangles) oocytes were negligible at all tested voltages. Cx43-G38E (open circles) expressing cells exhibited significantly increased peak currents at all voltages compared to either control or wild-type Cx43 oocytes. Data are the mean ± SE.

Single *Xenopus* oocytes were injected with RNAs encoding wild-type Cx43 or mutant Cx43-G38E to directly compare the levels of hemichannel activity. All oocytes were pre-injected with antisense oligonucleotides to inhibit the endogenous *Xenopus* Cx38 [40,41], which can also form active hemichannels [42]. Hemichannel currents were recorded by whole cell voltage clamp while the cells were stepped to different membrane voltages (Figure 2). Oocytes injected with either water, or wild-type Cx43 showed minimal whole cell membrane current when stepped to voltages between −30 and +60 mV. In contrast, oocytes injected with the Cx43-G38E mutant produced large whole cell currents upon depolarization, consistent with the presence of active connexin hemichannels [31,33,43].

To quantify the difference in hemichannel activity between wild-type Cx43 and the mutant Cx43-G38E, the peak mean currents (±SE) were plotted against the membrane potential. Both water injected control cells and wild-type Cx43 expressing cells showed negligible membrane currents at all tested membrane potentials between −30 and +60 mV. In contrast, the Cx43-G8V injected cells produced hemichannel currents that were increased 10 to 15 times in magnitude compared to either water, or wild-type Cx43, injected cells at all tested membrane potentials (*p* < 0.05, one way ANOVA). This significant increase in membrane current suggested the acquisition of augmented hemichannel activity by the Cx43-G38E mutant.

**Figure 3:**
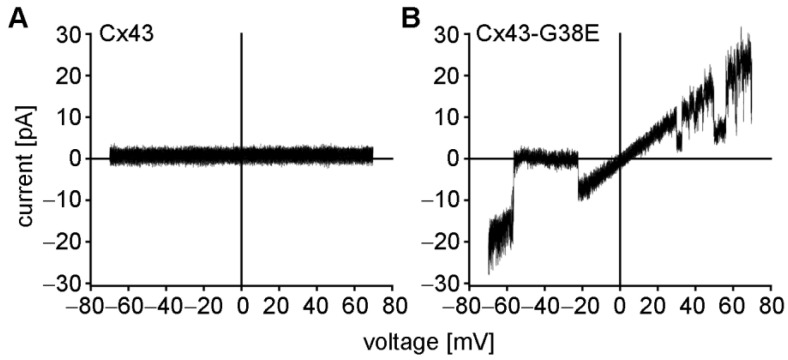
Patch clamp recordings from *Xenopus* oocytes injected with wild-type Cx43, or mutant Cx43-G38E RNAs. (**A**) Cell-attached patches obtained from wild-type Cx43 injected cells failed to display any single hemichannel activity between –70 and +70 mV. (**B**) Cx43-G38E expressing oocytes displayed single hemichannel currents that showed transitions to the fully closed state, or sub-conductance states.

Cell-attached patch clamp recordings were performed to confirm the presence of mutant Cx43-G38E hemichannels [30,31]. Cell-attached patches were obtained with a pipette solution containing 140 mM KCl and subjected to 8-s voltage ramps from −70 to +70 mV (Figure 3). Consistent with the lack of whole cell currents described above, wild-type Cx43 failed to form ion-conducting hemichannels in this voltage range, as has been previously reported [31,44]. In contrast, oocytes expressing mutant Cx43-G38E exhibited single hemichannel currents that transitioned from fully open to fully closed, or sub-conductance states. The current-voltage relation for Cx43-G38E was linear, with a unitary conductance of ~250 pS. Taken together, the data presented in Figure 2 and Figure 3 document a significant increase in hemichannel activity of the Cx43-G38E mutation when compared to wild-type Cx43.

### 2.3. The Cx43-G38E Mutation Localized to Cellular Interfaces in Transfected HeLa Cells

**Figure 4:**
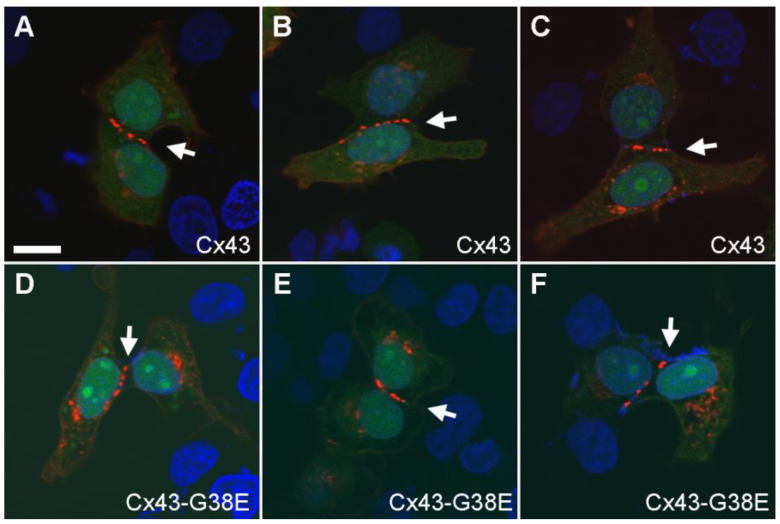
Localization of Cx43-G38E in transfected HeLa cells. (**A**–**C**) HeLa cells (DAPI stain, blue) transfected with wild-type Cx43 displayed a strong Cx43 antibody labeling (red) that concentrated in linear arrays of plaques at cell-to-cell interfaces (white arrowheads) and correlated with GFP fluorescence (green). (**D**–**F**) HeLa cells transfected with the Cx43-G38E mutation showed similar punctate Cx43 staining at cellular interfaces. Scale bar = 5 µm.

To examine whether the Cx43-G38E mutant protein localized at cell-to-cell interfaces in mammalian cells, connexin deficient HeLa cells were transiently transfected with the mutant, or wild-type forms of Cx43 using the pIRES2-EGFP vector (Figure 4). As previously reported, the culture media calcium concentration was increased to 2 mM by the addition of CaCl_2_ one day after transfection to enhance cell survival [31]. GFP expression (green) identified transfected cells. Immunostaining with antibodies against Cx43 (red) verified both protein expression and localization at cell-cell interfaces for wild-type Cx43 (Figure 4A–C) and Cx43-G38E (Figure 4D–F). Both wild-type Cx43 and the Cx43-G38E mutation accumulated in membrane regions of cell-to-cell contact, as shown by the linear areas of punctate staining (white arrows). Cell nuclei were stained with DAPI (blue). Thus, the Cx43-G38E mutant was properly targeted to gap junctional areas in transfected mammalian cells.

### 2.4. The Cx43-G38E Mutation Forms Functional Gap Junction Channels That Display Altered Voltage Gating

**Figure 5:**
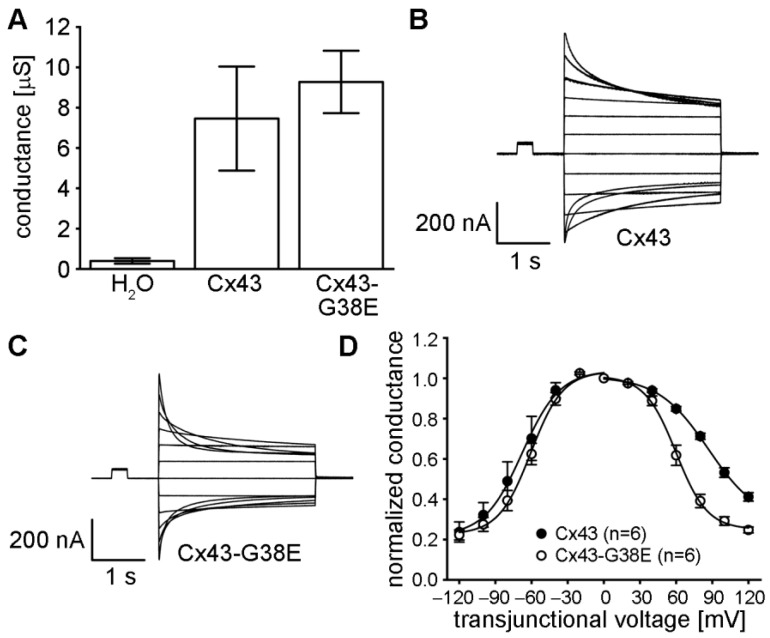
Cx43-G38E forms gap junction channels with altered voltage gating. (**A**) Gap junctional conductance was measured between *Xenopus* oocyte pairs injected with water (n = 16), wild-type Cx43 (n = 17), or mutant Cx43-38E (n = 22). Both wild-type and mutant Cx43 expressing cells were coupled at significantly greater values than the water injected controls (*p* < 0.05, one way ANOVA), but were not significantly different from each other (*p* > 0.05, *t*-test). (**B**) Wild-type Cx43 gap junction channels had junctional currents that decreased asymmetrically at higher trans-junctional potentials. (**C**) Junctional currents between cell pairs expressing Cx43-G38E displayed a more symmetrical decline. (**D**) Steady-state voltage gating of wild-type Cx43 (filled circles) showed an asymmetric decline in steady state conductance at increasing values of transjunctional voltage. Cell pairs expressing Cx43-G38E (open circles) had a more symmetrical reduction in conductance. Solid lines are Boltzmann fits of the data whose parameters are listed in Supplemental Table S1. Data are plotted as the mean ± SE.

The ability of the Cx43-G38E mutation to form functional gap junction channels was tested by expressing mutant and wild-type Cx43 in *Xenopus* oocyte pairs and measuring gap junctional conductance (Figure 5A). Oocyte pairs injected with water instead of connexin RNA displayed minimal junctional conductance (0.39 ± 0.13 µS, mean ± SE), whereas cells expressing wild-type Cx43 produced a mean conductance of 7.5 ± 2.6 µS. Cell pairs injected with the Cx43-G38E mutation had a mean conductance of 9.3 ± 1.5 µS. The conductance levels of cell pairs expressing either wild-type or mutant Cx43 were significantly larger than the water injected negative controls (one way ANOVA, *p* < 0.05). The difference in conductance between wild-type Cx43 and mutant Cx43-G38E was not statistically significant (paired *t*-test, *p* > 0.05). These data showed that the Cx43-G38E mutation formed gap junction channels with macroscopic conductance level equal to that of wild-type Cx43.

We next examined whether gap junction channels formed by the Cx43-G38E mutation had altered voltage gating properties compared to wild-type Cx43. Oocyte pairs expressing wild-type Cx43, or Cx43-G38E, were subjected to ±120 mV trans-junctional voltage potentials in 20 mV steps while recording the resulting junctional currents. As has been reported previously [45,46,47], junctional currents of wild-type Cx43 channels decreased in a voltage-dependent manner, with a slight asymmetry, showing less decay at positive values of trans-junctional potential (Figure 5B). In contrast, Cx43-G38E channels showed a more symmetric decline in junctional current at either polarity of trans-junctional potential (Figure 5C). This difference was confirmed and quantified by plotting the mean steady-state conductance (normalized to its values at ±20 mV) against the trans-junctional voltage (Figure 5D). Gap junction channels formed of wild-type Cx43 showed a slightly asymmetric decline in steady state conductance at increasing values of V_j_, as has been previously reported in the literature [35,46,48]. In contrast, channels from oocyte pairs expressing Cx43-G38E had steady state gating that was much more symmetrical than that of wild-type Cx43. Fitting of the steady state data to a Boltzmann equation [49] confirmed the increased symmetry, and parameters of those fits are provided in Supplemental Table S1. These data demonstrated that the voltage gating properties of gap junction channels made from Cx43-G38E differed quantitatively from those of wild-type Cx43.

## 3. Discussion

We have expressed and functionally characterized a dominant human Cx43 mutation, Cx43-G38E, which causes familial hypotrichosis, follicular keratosis and hyperostosis. We found that Cx43-G38E localized to gap junctional plaques in areas of cell apposition and formed gap junctional channels as efficiently as wild-type Cx43. Cx43-G38E gap junction channels displayed altered voltage gating compared to wild-type Cx43. In addition, Cx43-G38E formed functional hemichannels that facilitated a significantly increased magnitude of membrane current. In contrast, wild-type Cx43 failed to form ion-conducting hemichannels under the tested conditions, as has been reported previously [31,44,50,51,52]. This functional analysis of Cx43-G38E revealed that increased hemichannel function was a principal difference between wild-type Cx43 and a human Cx43 mutation linked to a unique skeletal-cutaneous phenotype.

The Cx43-G38E mutation was identified in a family where a mother and son presented with palmar keratoderma, total leukonychia, partial alopecia and hyperkeratotic plaques in friction zones [39]. These epidermal pathologies overlap considerably with the dermatological phenotype of human patients carrying the Cx43-E227D, or Cx43-A44V mutations causing erythro-keratodermia variabilis et progressive (EKVP) [26], or patients carrying the Cx43-G8V mutation causing palmoplantar keratoderma and congenital alopecia-1 (PPKCA1) [27]. All four of these mutations share a common gain of hemichannel function [27,30,31], suggesting that this attribute could contribute to the epidermal pathology shared by these disorders. However, Cx43-G38E patients also presented with hyperostosis of the skull and spine [39], which had not been described in EKVP or PPKCA1 patients. Wild-type Cx43 hemichannel activity has been implicated in normal bone development and function [53,54,55], so it is possible that the augmented hemichannel activity of Cx43-G38E could also disrupt bone homeostasis, leading to hyperostosis. Future experimentation into the precise functional differences between Cx43-G38E hemichannels and those formed by mutations causing EKVP or PPKCA1 could help differentiate between the hemichannel functions that cause skin disease and those that contribute to bone disorders.

Functional analysis of mutations in other connexin genes that have been associated with dermatological syndromes has suggested a general role for augmented hemichannel function in the pathology of skin disease [9,12,34]. Increased hemichannel activity has been reported for Cx26 mutations causing palmoplantar keratoderma (PPK) with deafness, or keratitis-ichthyosis-deafness (KID) syndrome [33,35,56,57]. In addition, Cx31 mutations linked to EKVP displayed increased hemichannel activity [58], as did Cx30 mutations causing hidrotic ectodermal dysplasia (HED) [32]. Recent studies in mouse models of KID and HED have shown that therapeutic strategies aimed at blocking hemichannel activity show promise as novel treatment paradigms for connexin mediated skin disease [13,14,15,59]. Whether this approach will show promise for treating the skeletal-cutaneous phenotype of Cx43-G38E patients or the other Cx43 mediated epidermal disorders will require further experimentation.

Increased hemichannel activity has also been documented as a consequence of mutations in other human connexin genes that cause congenital disorders. For example, cataract causing mutations in the GJA8 or GJA3 genes (encoding Cx50 and Cx46) have been show to produce increased hemichannel activity, suggesting that leaky hemichannels may cause cytotoxicity in the lens that leads to cataract development [60,61]. Even somatic mutations in connexin genes have implicated increased hemichannel activity in some vascular disorders. A somatic mutation in the GJA4 gene (Cx37-G41C) was found in >96% of patients with Orbital cavernous venous malformation (OCVM), a sporadic vascular anomaly characterized by dilated vascular channels. Expression of Cx37-G41C in Xenopus oocytes resulted in increased hemichannel activity and cell death [17]. Thus, our results from studying Cx43 mutations that augment hemichannel activity may provide functional insights into other connexinopathies [62].

Hyperactive connexin hemichannels were first linked to genetic skin disease in 2004 [57]. Years of ensuing research have reinforced a central role for aberrant hemichannel activity in human epidermal pathology and led to the point where novel therapies can be developed from mechanistic data on changes in channel function [63]. With the data on Cx43-G38E presented here, four different Cx43 mutations shown to form aberrant hemichannels have been linked to prominent epidermal pathology in humans [27,30,31]. Future studies would benefit from mouse models of the Cx43 mediated skin disorders. These could be used to both validate the role of Cx43 hemichannel activity in disease progression and to facilitate testing of hemichannel inhibitors as possible therapeutic tools. Hemichannel dysfunction is likely to contribute to pathological mechanisms in many of the different connexinopathies [8,59,64,65], highlighting the need for continued research on the functional consequences of connexin mutations that cause human genetic disease.

## 4. Materials and Methods

### 4.1. Molecular Cloning

The human Cx43 coding sequence was cloned into pCS2^+^ [66] for functional studies in *Xenopus* oocytes as previously described [35]. The Cx43-G38E mutant was generated by site directed mutagenesis [67] using wild-type human Cx43 as a template. Cx43-G38E was then cloned into pBlueScript II (Agilent Technologies, Santa Clara, CA, USA) and sequenced on both strands prior to being subcloned into pCS2+ for *Xenopus* oocyte expression, or pIRES2-EGFP2 (Clontech Laboratories, Mountain View, CA, USA) for expression in HeLa cells [43].

### 4.2. In Vitro Transcription and Xenopus Oocyte Preparation

Wild-type and mutant Cx43 constructs in pCS2^+^ were linearized with Not1 and cRNA was transcribed using the SP6 mMessage mMachine (Ambion, Austin, TX, USA). Wild-type and mutant RNA samples were prepared in parallel and RNA yields and purity were assessed by reading the ultraviolet absorbance at 260 and 280 nm. *Xenopus laevis* oocytes were purchased (Xenopus 1, Dexter, MI, USA) and cultured in modified Barth’s (MB) medium [31,43]. Oocytes were first injected with 10 ng of an antisense oligonucleotide against the endogenous *Xenopus* Cx38 [40,41], followed by connexin transcripts (5 ng/cell). Antisense Cx38 oligonucleotide treated oocytes injected with water, instead of cRNA, were used as negative controls. For measurements of gap junctional conductance, *Xenopus* oocytes were manually devitellinized in a hypertonic solution consisting of (in mM) 220 Na-aspartate, 10 KCl, 2 MgCl_2_ and 10 HEPES and the oocytes were paired with their vegetal poles in direct contact. Prior to recording, injected oocytes were incubated at 18 °C in MB medium supplemented with 2 mM Ca^2+^ to maintain viability [33,68].

### 4.3. Hemichannel Current Recording

Whole cell wild-type and mutant Cx43 hemichannel currents were recorded 24 h after cRNA injection using a GeneClamp 500 amplifier operated by a PC-compatible computer using a Digidata 1440 A interface and pClamp 10 software (Axon Instruments, Foster City, CA, USA). Electrodes (1.5 mm diameter glass, World Precision Instruments, Sarasota, FL) were pulled to a resistance of 1–2 MΩ (Narishige, Tokyo, Japan) and filled with 3 M KCl, 10 mM EGTA and 10 mM HEPES, pH 7.4. All hemichannel currents were recorded in MB medium without added calcium [33]. Current-voltage (I–V) curves were generated by clamping cells at −40 mV and imposing voltage steps ranging from −30 to +60 mV in 10 mV increments.

Patch clamp recording of single-hemichannel currents were obtained from *Xenopus* oocytes that had been manually devitellinized and then placed in MB medium supplemented with 2 mM Ca^2+^ in agarose coated dishes for recovery. Individual oocytes were moved to a recording chamber (RC-28; Warner Instruments) filled with the same solution as the patch pipette, composed of (in mM): 140 KCl, 1 MgCl2, 5 HEPES, 1 CaCl2 and 3 EGTA, pH adjusted to 8.0 with KOH. The bath was connected to a ground chamber containing the same solution. Recordings of single hemichannel currents from voltage ramps ranging from −70 to +70 mV were leak subtracted as previously described [31].

### 4.4. Measurement of Gap Junctional Conductance

Pairs of *Xenopus* oocytes were used to measure gap junctional conductance (G_j_), by clamping both cells in a pair initially at −40 mV (zero trans-junctional potential, or V_j_). One cell was then alternately pulsed ±20 mV and the current produced after the change in voltage was recorded in the second cell. This current equaled the junctional current (I_j_) in magnitude. Values of G_j_ were obtained in each tested cell pair by dividing I_j_ by the voltage difference, G_j_ = I_j_/(V1 − V2) [49]. To determine voltage gating properties, the junctional current was recorded in response to hyperpolarizing or depolarizing V_j_s in 20-mV steps. Steady state current (I_jss_) values were recorded at the end of the voltage pulse. To calculate the steady state conductance (G_jss_), I_jss_ was divided by V_j_, normalized to the values obtained at ±20 mV and plotted against V_j_. Data were fit to a Boltzmann relation:

G_jss_ = (G_jmax_ − G_jmin_)/(1 + exp [A (V_j_ − V_0_)]) + G_jmin_ (1)

where G_jmax_ was the maximum conductance, G_jmin_ is the residual conductance and V_0_ is the trans-junctional voltage at which G_jss_ = (G_jmax_ − G_jmin_)/2. A (=*n*q/kT) represents the number (*n*) of electron charges (q) moving through the membrane where k is the Boltzmann constant and T is the absolute temperature.

### 4.5. Western Blotting

*Xenopus* oocytes were lysed and membrane protein extracts were prepared as described previously [69], separated by 12% SDS-PAGE and transferred to nitrocellulose. Western blots were blocked with 5% milk, 0.1% Tween20 in TBS and then initially probed with polyclonal antibodies against Cx43 (Life Technologies, Carlsbad, CA, USA). After washing, blots were probed with horseradish peroxidase conjugated secondary antibodies (Jackson Laboratories and GE Healthcare) and visualized with chemiluminescence. Blots were then stripped and re-probed with a monoclonal anti-β-tubulin antibody (Abcam, Cambridge, MA, USA) as a loading control. ImageJ software version 1.49v [70] was used to determine band densities from five independent experiments.

### 4.6. Cell Transfection and Immunofluorescent Staining

HeLa cells were grown on coverslips to 50% confluence and transfected with wild-type Cx43 or mutant Cx43-G38E using Lipofectamine 2000 (Invitrogen, Carlsbad, CA, USA) as previously described [35,71,72]. To improve cell survival, the culture media calcium concentration was increased to 2 mM by the addition of CaCl_2_ one day after transfection [31]. HeLa cells were fixed in 1% paraformaldehyde in PBS two days after transfection and blocked with 5% BSA, 0.02% NaN_3_ and 0.1% Tx-100 in PBS. Cells were stained with a polyclonal anti-Cx43 antibody followed by a Cy3-conjugated goat anti-rabbit secondary antibody (Jackson ImmunoResearch, West Grove, PA, USA). Coverslips were mounted on microscope slides and photographed using a DP72 digital camera on a BX51 microscope (Olympus America, Waltham, MA, USA).

### 4.7. Statistical Analysis

Differences between data sets were analyzed for statistical significance using Origin 2020 software (Microcal Software, Northampton, MA, USA). Statistical significance was designated for analyses with *p* values < 0.05.

## Data Availability

Not applicable.

## Supplementary Materials

The following supporting information can be downloaded at: https://www.mdpi.com/article/10.3390/ijms24032222/s1.
