# Supplementary material for: Increased Hemichannel Activity Displayed by a Connexin43 Mutation Causing a Familial Connexinopathy Exhibiting Hypotrichosis with Follicular Keratosis and Hyperostosis
Source: Int J Mol Sci. 2023 Jan 22;24(3):2222. doi: 10.3390/ijms24032222 (PMC9916973; doi:10.3390/ijms24032222)
Supplement: Supplementary file 1 [file ijms-24-02222-s001.zip › ijms-2139873-supplementary.pdf]

Supplementary Table S1. Boltzmann parameters* for wild-type Cx43 and mutant Cx43-G38E gap junction channels.

Cell Injection	V_j	V_0	G_{jmin}	A
wild-type Cx43	+	86.8	0.29	0.048
wild-type Cx43	-	67.8	0.22	0.064
mutant Cx43-G38E	+	59.9	0.25	0.077
mutant Cx43-G38E	-	60.7	0.23	0.075

* V_j is the transjunctional membrane potential, with + and - indicating polarity. V_0 indicates the voltage measured midway through the junctional conductance decline (in mV), G_{jmin} represents the minimum junctional conductance value, and A denotes the cooperativity constant, which reflects the number of charges moving through the transjunctional field.
